# Predictors of Lymphoceles in Women Who Underwent Laparotomic Retroperitoneal Lymph Node Dissection for Early Gynecologic Cancer: A Retrospective Cohort Study

**DOI:** 10.3390/ijerph16060936

**Published:** 2019-03-15

**Authors:** Hui-Hua Chen, Wan-Hua Ting, Ho-Hsiung Lin, Sheng-Mou Hsiao

**Affiliations:** 1 Department of Obstetrics and Gynecology, Far Eastern Memorial Hospital, Banqiao, New Taipei 220, Taiwan; thandaaye24@gmail.com (H.-H.C.); stellatingwh@yahoo.com (W.-H.T.); hhlin@ntuh.gov.tw (H.-H.L.); 2 Department of Obstetrics and Gynecology, National Taiwan University College of Medicine and the Hospital, Taipei 100, Taiwan; 3 Graduate School of Biotechnology and Bioengineering, Yuan Ze University, Taoyuan 320, Taiwan

**Keywords:** lymph node excision, lymphocele, peritoneum, drainage, gynecology

## Abstract

*Background:* Lymphoceles could represent a detrimental complication after retroperitoneal lymph node dissection. Our aim was to elucidate predictors of lymphoceles. *Methods:* Between 2011 and 2017, medical records of consecutive women who underwent laparotomic retroperitoneal lymph node dissection for FIGO stage I or II gynecologic cancer were reviewed. *Results:* A total of 204 women, including those with lymphoceles (n = 31) and symptomatic lymphoceles (n = 7), were reviewed. According to multivariable analysis, parity (odds ratio = 0.59, *p* = 0.003), adjuvant pelvic radiotherapy (odds ratio = 2.60, *p* = 0.039), and peritoneal nonclosure without pelvic drainage (odds ratio = 2.31, *p* = 0.048) were predictors of lymphoceles. In addition, parity (odds ratio = 0.73, *p* = 0.03), hypertension (odds ratio = 2.62, *p* = 0.02), and peritoneal partial closure with pelvic drainage (odds ratio = 0.27, *p* = 0.02) were predictors of complications. *Conclusion:* Low parity, adjuvant pelvic radiotherapy, and peritoneal nonclosure without pelvic drainage were associated with increased lymphocele formation. In addition, a lower complication rate was found in the peritoneal partial closure with pelvic drainage group; thus, peritoneal partial closure with pelvic drainage might be suggested for women who undergo laparotomic retroperitoneal lymph node dissection.

## 1. Introduction

Retroperitoneal lymph node dissection is included in the treatment of oncogynecological operative procedure. One of the common complications of retroperitoneal lymph node dissection is the formation of lymphoceles.

Retroperitoneal lymph node dissection would result in disruption of lymphatic tracts and leakage of lymphatic fluid. Lymphoceles are lymphatic fluid collections in retroperitoneal space and were first described in 1955 [1]. The incidence of lymphocele formation after retroperitoneal lymph node dissection ranges from 1 to 58% [1,2,3,4,5]. Most lymphoceles are asymptomatic. They may resolve spontaneously with the development of new lymphatic vessels [6]. However, 5 to 34.5% of lymphoceles are symptomatic [5,7,8,9,10]. Symptoms of lymphoceles are usually related to compression to adjacent organs, infection, or fistula. Symptomatic lymphoceles usually need to be treated, and may result in a delay in postoperative adjuvant therapy. Thus, it is important to minimize the incidence of lymphoceles.

Following pelvic lymph node dissection, different surgical techniques have been suggested to prevent surgery-related morbidity, such as peritoneal nonclosure and implantation of a retroperitoneal suction drainage catheter.

Peritoneal closure has been recommended after pelvic and/or para-aortic lymph node dissection; however, more recently, several studies have shown that peritoneal closure could elevate the risk of lymphocele formation [9,10,11]. It has been suggested that the peritoneum should be left open to allow the lymphatic fluid flows into the peritoneal cavity and be absorbed [12,13]. However, some studies did not find increased lymphoceles after peritoneal closure compared with nonclosure [14,15].

In addition, pelvic retroperitoneal drainage catheter insertion is recommended to prevent lymphocele formation [16]. However, Charoenkwan et al. reported that tube drain placement is associated with a higher risk of short- and long-term symptomatic lymphocele formation [17]. Franchi et al. reported that there was no difference in the lymphocele rate between the drainage and nondrainage groups [18]. We were interested in which procedures would decrease lymphocele formation. 

Besides, systemic lymphadenectomy did not improve overall survival in patients with optimally debulked advanced ovarian cancer [19], and we did not routinely perform systemic lymphadenectomy in patients with advanced ovarian cancer. Therefore, only patients with early gynecologic cancer were enrolled in this study. Thus, the aim of this study was to evaluate predictors of lymphoceles in early gynecologic cancer patients who underwent laparotomic retroperitoneal lymph node dissection.

## 2. Materials and Methods

The medical records of women with a Federation of Gynecologic and Obstetrics (FIGO) stage I or II gynecological cancer, including cervical cancer, endometrial cancer, ovarian cancer, and fallopian tubal cancer, who underwent laparotomic retroperitoneal lymph node dissection from January 2011 to December 2017 in Far Eastern Memorial Hospital, a tertiary referral center, were reviewed. Far Eastern Memorial Hospital Research Ethics Review Committee approved the protocol (No: FEMH-107150-E; ClinicalTrials.gov Identifier: NCT03765320, date of registration: 5December2018).

Retroperitoneal lymph node dissection was defined as the removal of lymphatic tissue in the pelvic regions with or without para-aortic lymph node dissection. The choice of peritoneal closure methods, including complete closure, partial closure (i.e., the peritoneum was closed by interrupted sutures with open space ~3 cm between sutures), and nonclosure, was based on each surgeon’s preference. The decision of retroperitoneal suction drainage was also dependent on each surgeon’s preference. De novo abdominal or pelvic cysts in ultrasonography, computed tomography, or magnetic resonance imaging after para-aortic and/or pelvic lymphadenectomy were considered to have lymphoceles. Additionally, perioperative complications, adjuvant chemotherapy, adjuvant radiotherapy, and clinical oncologic outcome were also reviewed. Parity was determined by the numbers of pregnancies reaching 20 weeks and not by the number of fetuses delivered [20].

The STATA software program (Version 11.0; Stata Corp, College Station, TX, USA) was used for statistical analyses. Chi-square test, Fisher’s exact test, Wilcoxon rank-sum test, or Spearman’s rank-correlation coefficient were employed for statistical analysis. Multivariable backward stepwise analysis was performed using all variables in the univariate analysis until all remaining variables became significant. A *p*-value of less than 0.05 was considered statistically significant.

## 3. Results

Two-hundred-and-four women who underwent laparotomic surgery and retroperitoneal lymph node dissection for early gynecologic cancers (i.e., stage I or II gynecologic cancer) were included in this study. The baseline data of the women are listed in Table 1. All patients underwent pelvic lymph node dissection, and 127 (62.3%) patients underwent concomitant para-aortic lymph node removal. All patients in the peritoneal complete closure group (n = 44) and 44 of 45 patients in the peritoneal partial closure group received concomitant pelvic drainage. Nonetheless, only 24 patients in the peritoneal nonclosure group (n = 115) received concomitant pelvic drainage. 

Among the 204 women, thirty-one (15.1%) patients had lymphoceles. Symptomatic lymphoceles, including compression symptoms (n = 4) and infection (n = 3), were found in seven women. A variety of treatments, including computerized tomographic guided drainage (n = 4), laparoscopic marsupialization (n = 1), aspiration (n = 1), and intravenous antibiotics (n = 1), were used to treat patients with symptomatic lymphoceles.

Comparisons of the incidences of lymphoceles and complications were tabulated in Table 2. There were significant between-group differences in the body mass index and the complication rates (Table 2). Nonetheless, multivariable backward stepwise logistic analysis was performed using all variables in the univariate analysis to predict lymphoceles. Parity (odds ratio = 0.59, *p* = 0.003), adjuvant pelvic radiotherapy (odds ratio = 2.60, *p* = 0.039), and peritoneal nonclosure without pelvic drainage (odds ratio = 2.31, *p* = 0.048) were predictors of lymphoceles (Table 3).

**Figure 1:**
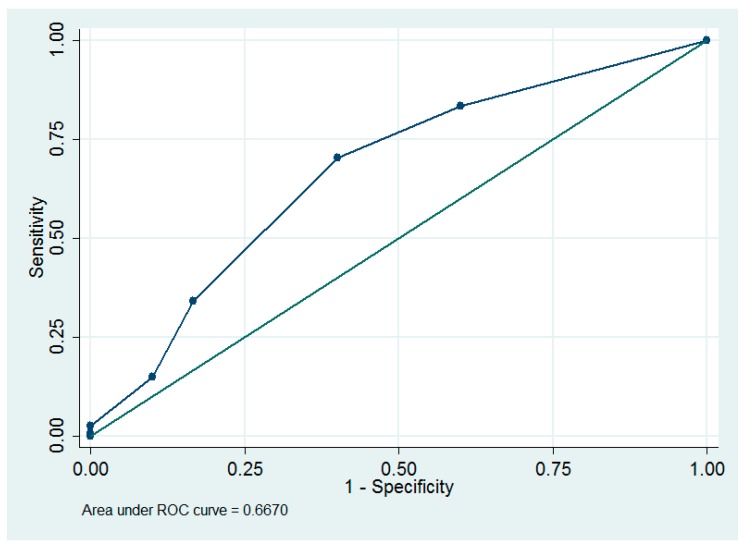
The receiver operating characteristic (ROC) curves of using parity to predict lymphoceles.

The optimum cut-off value of parity ≤2 to predict lymphoceles was determined using receiver operating characteristic (ROC) curve analysis, which has an area under the ROC curve of 0.67 (95% confidence interval (CI) = 0.56 to 0.78; sensitivity = 70.4%, specificity = 60.0%, Figure 1). 

Complications included lymphoceles, postoperative ileus, and miscellaneous (Table 1). Multivariable backward stepwise logistic analysis was performed using all variables in the univariate analysis to predict complications. Parity (odds ratio = 0.73, *p* = 0.03), hypertension (odds ratio = 2.62, *p* = 0.02), and peritoneal partial closure with pelvic drainage (odds ratio = 0.27, *p* = 0.02) were predictors of complications (Table 4).

**Figure 2:**
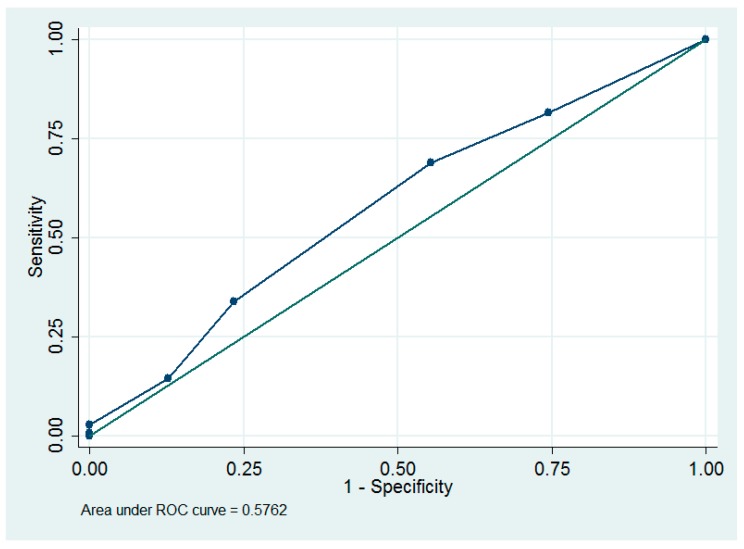
The receiver operating characteristic (ROC) curves of using parity to predict complications.

The optimum cut-off value of parity ≤2 to predict complications was determined using ROC curve analysis, which has an area under the ROC curve of 0.58 (95% CI = 0.48 to 0.67; sensitivity = 69.0%, specificity = 44.7%, Figure 2).

In addition, cancer recurrence was not associated with peritoneal partial closure with pelvic drainage, pelvic drainage, or presence of lymphoceles (all *p* > 0.05, Table 5).

## 4. Discussion

In our study, peritoneal nonclosure without pelvic drainage was associated with increased lymphocele formation (odds ratio = 2.31, *p* = 0.048, Table 3). Similarly, Bafna et al. found that peritoneal partial closure without pelvic drainage was associated with increased lymphocele formation (28%) [15]. In contrast, some authors observed a lower incidence of lymphoceles in the peritoneal nonclosure/partial closure group compared with the closure group [12,13,21,22]. In addition, some studies reported that the incidence of lymphoceles did not differ between the peritoneal nonclosure groups and the peritoneal closure group [14,15]. Thus, whether peritoneal nonclosure could increase or decrease lymphocele formation remains undetermined. 

Retroperitoneal drainage with catheter insertion is traditionally recommended to remove leaking lymph and thus prevent lymphocele formation [11]. We also found that peritoneal nonclosure without pelvic drainage was associated with increased lymphocele formation (odds ratio = 2.31, *p* = 0.048, Table 3). Nonetheless, in the Cochrane review, retroperitoneal drainage did not have benefit for preventing lymphocele formation [17], and peritoneal nonclosure with pelvic drainage was associated with a higher risk of symptomatic lymphocele formation [17]. Franchi et al. and Lopes et al. also reported that there was no difference in the incidence of lymphocele formation between the drainage and nondrainage groups [18,23]. However, from our study, peritoneal nonclosure with pelvic drainage (odds ratio = 1.13, *p* = 0.83, Table 3) was not a predictor for lymphocele formation; thus, it might be better to implant a pelvic drainage tube if the peritoneum is not closed. 

In our study, adjuvant pelvic radiotherapy were associated with increased lymphocele formation (odds ratio = 2.60, *p* = 0.039, Table 3). Petru et al. also found a greater incidence of lymphoceles in cervical cancer patients who underwent postoperative adjuvant radiotherapy and/or chemotherapy compared with no adjuvant therapy (60% vs. 32.6%, respectively, *p* = 0.01) [9], and a more advanced disease might explain the finding of a greater incidence of lymphoceles in the patients receiving adjuvant therapy [9]. However, in our study, we included cancer stage as one of the variables in multivariable backward stepwise logistic analysis (Table 3); thus, adjuvant radiotherapy as a predictor for lymphocele formation should be less likely related to more advanced disease. Instead, we speculate that adjuvant pelvic radiotherapy might be detrimental to the development of new lymphatic vessels, thus subsequently resulting in delayed absorption of lymph and a greater incidence of lymphocele formation found during the follow-up.

In our study, increased parity was associated with decreased lymphocele formation (odds ratio = 0.59, *p* = 0.003, Table 3). It was reported that the number of parity was significantly correlated with microvessel counts, and this might be due to the effects of hormone stimulation during previous pregnancies [24]. Thus, we speculate that parity might be also positively correlated with lymphatic microvessels, and this may explain the positive correlation between increased parity and decreased lymphocele formation. In our study, we found that parity ≤2 was an optimum cut-off value to predict lymphoceles and complications. We can use the above cut-off value as a guideline for pretreatment consultation. However, owing to their low areas under the ROC curve (Figure 1, Figure 2), parity is not a good predictor of lymphoceles and complications.

Franchi et al. reported that the infection and adhesion complication rates did not differ between the peritoneal closure and nonclosure groups [14]. However, in our study, peritoneal partial closure with pelvic drainage was associated with a lower complication rate (odds ratio = 0.27, *p* = 0.02, Table 4). Thus, partial peritoneal closure with pelvic drainage might be suggested for women who underwent retroperitoneal lymph node dissection. Zikan et al. reported that radical hysterectomy is an independent predictor for symptomatic lymphoceles (odds ratio = 2.208, 95% CI = 1.058 to 4.606, *p* = 0.035) [25]. In Franchi et al.’s study, all patients received either peritoneal complete closure or nonclosure, and underwent radical hysterectomy and pelvic drainage [14]. However, 45 (22%) of our patients underwent peritoneal partial closure, 45 (22%) of our patients underwent radical hysterectomy, and 112 (55%) of our patients underwent pelvic drainage (Table 1). Thus, the difference in the peritoneal closure, patient composition, and pelvic drainage between our and Franchi et al.’s studies might partly explain the between-group difference in the finding of peritoneal partial closure with pelvic drainage as a predictor of low complication rate.

In this study, hypertension was a predictor for complications (odds ratio = 2.62, *p* = 0.02, Table 4). Despite of statistical insignificance, our patients with hypertension had higher rates of postoperative ileus (3/56 vs. 6/148, *p* = 0.71) and surgical wound infection/dehiscence (3/56 vs. 2/148, *p* = 0.13), compared with patients without hypertension. Similarly, Sheyn et al. reported that a higher incidence of hypertension was found in patients with small bowel obstruction, compared with patients without small bowel obstruction (37.1% vs. 27.3%, *p*< 0.001) [26]. Kaneko et al. reported that hypertension was a risk factor for incisional hernia in patients underwent loop ileostomy closure (odds ratio = 2.60, 95% CI = 1.01 to 6.69, *p* = 0.046) [27]. Hypertension has been found to promote abnormal wound healing by inducing endothelial dysfunction, aggravate the inflammation-induced hypoxia, and aggravate pathological skin fibroblast behavior [28]. 

Eighteen (8.8%) patients had cancer recurrence (Table 1). However, we did not find any association between lymphocele formation and cancer recurrence (Table 5). Besides, we did not find any literature that reported a significant association between lymphocele formation and cancer recurrence.

The limitations of this study include the retrospective nature, not a randomized trial, and a limited sample size. Further prospective randomized studies should be performed to confirm our findings. In addition, nearly all our patients in the peritoneal complete and partial closure groups had concomitant pelvic drainage. Thus, our conclusion should not be generalized for those who underwent pelvic peritoneal closure without pelvic drainage.

## 5. Conclusions

Low parity, adjuvant pelvic radiotherapy, and peritoneal nonclosure without pelvic drainage were associated with increased lymphocele formation. In addition, a lower complication rate was found in the peritoneal partial closure with pelvic drainage group; thus, peritoneal partial closure with pelvic drainage might be suggested for women who undergo laparotomic retroperitoneal lymph node dissection.

## Figures and Tables

**Table 1:** Baseline data of women who underwent laparotomic surgery for early gynecologic cancers (n = 204).

Variables	Values
Age (years)	53.3 ±10.6
Parity	1.9 ± 1.4
Body mass index (kg/m^2^)	26.0 ± 5.8
Ovarian cancer or borderline tumor (stage I, n = 56; stage 2, n = 13)	69 (34)
Endometrial cancer (stage I, n = 84; stage 2, n = 7)	91 (45)
Cervical or vaginal cancer (stage I, n = 41; stage 2, n = 3)	44 (22)
Diabetes	24 (12)
Hypertension	56 (27)
Peritoneal complete closure with pelvic drainage	44 (22)
Peritoneal partial closure with pelvic drainage	44 (22)
Peritoneal partial closure without pelvic drainage	1 (1)
Peritoneal nonclosure with pelvic drainage	24 (12)
Peritoneal nonclosure without pelvic drainage	91 (45)
Pelvic drainage	112 (55)
Adjuvant pelvic radiotherapy	43 (21)
Adjuvant vaginal brachytherapy	31 (15)
Adjuvant chemotherapy	75 (34)
Complications	50 (12)
Lymphocyst	31 (14.7)
Symptomatic lymphocyst	7 (3.4)
Postoperative ileus	9 (4.4)
Miscellaneous	11 (5.3)
Recurrence of cancer	18 (8.8)

Data were expressed as the mean ± standard deviation or number (percentage).

**Table 2:** Subgroup comparisons of baseline data, rates of lymphoceles and complications (n = 204).

Variables	Peritoneal Complete Closure with Pelvic Drainage (a, n = 44)	Peritoneal Partial Closure with Pelvic Drainage (b, n = 44)	Peritoneal Partial Closure without Pelvic Drainage (c, n = 1)	Peritoneal Nonclosure with Pelvic Drainage (d, n = 24)	Peritoneal Nonclosure without Pelvic Drainage (e, n = 91)	† p	‡ Post Hoc Analysis
Age (years)	52.6 ± 10.8	53.7 ± 11.1	-	53.9 ± 8.3	53.4 ± 11.0	0.98	
Parity	2.1 ± 1.5	1.9 ± 1.4	-	1.6 ± 1.2	1.9 ± 1.3	0.68	
Body mass index (kg/m^2^)	26.7 ± 5.4	28.0 ± 7.5	-	23.2 ± 4.8	25.3 ± 4.9	0.01	b vs. c, p = 0.001
Diabetes							
Yes	2 (5)	9 (20)	0 (0)	2 (9)	11 (12)	0.21	
No	42 (95)	35 (80)	1 (100)	22 (91)	80 (88)		
Hypertension							
Yes	8 (18)	14 (32)	0 (0)	4 (17)	30 (33)	0.23	
No	36 (82)	30 (68)	1 (100)	20 (83)	61 (67)		
Stage							
I	41 (93)	35 (80)	1 (100)	20 (83)	84 (92)	0.15	
II	3 (7)	9 (20)	0 (0)	4 (17)	7 (8)		
Lymphocele							
Yes	4 (9)	4 (9)	0 (0)	4 (17)	19 (21)	0.28	
No	40 (91)	40 (91)	1 (100)	20 (83)	72 (79)		
Complication							
Yes	7 (16)	5 (11)	0 (0)	9 (38)	29 (32)	0.02	a vs. d, p = 0.045
No	37 (84)	39 (89)	1 (100)	15 (62)	62 (68)		b vs. d, p = 0.01 b vs. e, p = 0.01

Data were expressed as mean ± standard deviation or n (percentage). † Oneway ANOVA test or Fisher’s exact test. ‡ Chi-square test or Wicoxon rank-sum test.

**Table 3:** Univariate and multivariable backward stepwise logistic analysis to predict lymphoceles (n = 204).

Variables	Univariate Analysis		Multivariable Analysis	
	Odds Ratio (95% CI)	^†^ *p*	Odds Ratio (95% CI)	^‡^ *p*
Age (years)	0.99 (0.95–1.02)	0.53	-	-
Parity	0.62 (0.45–0.86)	0.004	0.59 (0.42–0.84)	0.003
Body mass index (kg/m^2^)	0.97 (0.91–1.05)	0.48	-	-
Diabetes	1.13 (0.36–3.58)	0.83	-	-
Hypertension	1.10 (0.47–2.55)	0.83	-	-
Ovarian cancer	1.09 (0.49–2.43)	0.83	-	-
Radical hysterectomy	0.82 (0.32–2.15)	0.69	-	-
Stage of cancer	0.82 (0.23–2.94)	0.76	-	-
Adjuvant chemotherapy	1.77 (0.82–3.82)	0.15	-	-
Adjuvant pelvic radiotherapy	2.42 (1.06–5.56)	0.04	2.60 (1.05–6.45)	0.039
Adjuvant vaginal brachytherapy	1.42 (0.53–3.81)	0.49	-	-
Peritoneal complete closure with pelvic drainage (n = 44)	0.49 (0.16–1.49)	0.21	-	-
Peritoneal partial closure with pelvic drainage (n = 44)	0.49 (0.16–1.48)	0.21	-	-
^§^ Peritoneal partial closure without pelvic drainage (n = 1)	-	-	-	-
Peritoneal nonclosure with pelvic drainage (n = 24)	1.13 (0.36–3.58)	0.83	-	-
Peritoneal nonclosure without pelvic drainage (n = 91)	2.22 (1.01–4.86)	0.046	2.31 (1.01–5.30)	0.048

Data were expressed as odds ratios (95% confidence intervals). CI = confidence interval. ^†^ Logistic regression analysis. ^‡^ Multivariate backward stepwise logistic analysis was performed using all variables in the univariate analysis until all remaining variables became significant. Pseudo R^2^ = 0.10. ^§^ Peritoneal partial closure without pelvic drainage predict no complication perfectly, thus logistic regression analysis cannot be performed for this variable.

**Table 4:** **Univariate** and multivariable backward stepwise logistic analysis to predict complications (n = 204).

Variables	Univariate Analysis		Multivariable Analysis	
	Odds Ratio (95% CI)	^†^ *p*	Odds Ratio (95% CI)	^‡^ *p*
Age (years)	1.02 (0.99–1.05)	0.19	-	-
Parity	0.82 (0.63–1.05)	0.11	0.73 (0.55–0.96)	0.03
Body mass index (kg/m^2^)	0.97 (0.91–1.03)	0.25	-	-
Diabetes	0.79 (0.28–2.24)	0.66	-	-
Hypertension	1.94 (0.98–3.83)	0.057	2.62 (1.18–5.81)	0.02
Ovarian cancer	1.14 (0.58–2.22)	0.71	-	-
Radical hysterectomy	1.34 (0.64–2.81)	0.44	-	-
Stage of cancer	1.40 (0.54–3.64)	0.49	-	-
Adjuvant chemotherapy	1.07 (0.56–2.07)	0.84	-	-
Adjuvant pelvic radiotherapy	1.45 (0.69–3.07)	0.33	-	-
Adjuvant vaginal brachytherapy	0.88 (0.36–2.19)	0.79	-	-
Peritoneal complete closure with pelvic drainage (n = 44)	0.51 (0.21–1.24)	0.14	-	-
Peritoneal partial closure with pelvic drainage (n = 44)	0.32 (0.12–0.88)	0.03	0.27 (0.09–0.83)	0.02
^§^ Peritoneal partial closure without pelvic drainage (n = 1)	-	-	-	-
Peritoneal nonclosure with pelvic drainage (n = 24)	2.03 (0.83–4.99)	0.12	-	-
Peritoneal nonclosure without pelvic drainage (n = 91)	2.05 (1.07–3.91)	0.03	-	-

Data were expressed as odds ratios (95% confidence intervals). CI = confidence interval. ^†^ Logistic regression analysis. ^‡^ Multivariate backward stepwise logistic analysis was performed using all variables in the univariate analysis until all remaining variables became significant. Pseudo R^2^ = 0.07. ^§^ Peritoneal partial closure without pelvic drainage predict no complication perfectly, thus logistic regression analysis cannot be performed for this variable.

**Table 5:** Correlation of cancer recurrence and peritoneal closure/drainage/lymphoceles (n = 204).

Variables	Spearman’s Rho	^†^ *p*
Peritoneal partial closure with pelvic drainage	−0.08	0.26
Pelvic drainage	−0.10	0.15
Presence of lymphoceles	−0.04	0.62

^†^ Spearman’s rank correlation test.
